# Mucins and mucinous ovarian carcinoma: Development, differential diagnosis, and treatment

**DOI:** 10.1016/j.heliyon.2023.e19221

**Published:** 2023-08-17

**Authors:** Yicong Wang, Lifeng Liu, Yongai Yu

**Affiliations:** Department of Obstetrics and Gynecology, Dalian Municipal Central Hospital, Dalian, China

**Keywords:** Mucin, Mucinous ovarian carcinoma, Tumorigenesis, Chemoresistance, Targeted therapy

## Abstract

Mucinous ovarian carcinoma (MOC) is a rare histological type of epithelial ovarian cancer. It has poor response to conventional platinum-based chemotherapy regimens and PARPi-based maintenance treatment, resulting in short survival and poor prognosis in advanced-disease patients. MOC is characterized by mucus that is mainly composed of mucin in the cystic cavity. Our review discusses in detail the role of mucins in MOC. Mucins are correlated with MOC development. Furthermore, they are valuable in the differential diagnosis of primary and secondary ovarian mucinous tumors. Some types of mucins have been studied in the context of chemoresistance and targeted therapy for ovarian cancer. This review may provide a new direction for the diagnosis and treatment of advanced MOC.

## 1 Introduction

Mucinous ovarian carcinoma (MOC) is a rare tumor that probably accounts for 3%–5% of all cases of epithelial ovarian cancer (EOC) [1,2]. For decades, the management of MOC was based on guidelines developed for serous ovarian cancer. However, MOC responds poorly to conventional platinum-based chemotherapy regimens and maintenance treatment based on poly ADP-ribose polymerase inhibitors（PARPi), which result in low survival rates and poor prognosis, particularly in patients with advanced-stage disease [3]. MOC is a unique disease requiring unique management with an understanding of its biological features. MOC is characterized by a large, multilocular neoplasm with mucus in the cystic cavity. The main components of mucus are glycoproteins, of which most are mucins. Studies have found that if the neoplasm of MOC ruptures preoperatively or intraoperatively, mucus in the cystic cavity may lead to an increased risk of long-term recurrence and metastasis [4]. Mucins have also been found to be associated with chemoresistance in mucinous carcinomas [4,5]. Therefore, mucins may be a potential therapeutic target for MOC. In the present review, we describe the clinical features and treatment challenges of MOC and the role of mucins in the development, differential diagnosis, and treatment of MOC. This review may provide a new direction for the diagnosis and treatment of advanced MOC.

## 2 Clinical features and treatment challenges of MOC

In general, 65%–80% of MOC cases are diagnosed in the early stage [6]. Patients with International Federation of Gynecology and Obstetrics (FIGO) stage I MOC have a 5-year overall survival rate that is close to 90% [7]. In contrast, patients with advanced MOC (FIGO stages II–IV) have a much worse prognosis than patients with advanced serous ovarian cancer at similar stages treated with comparable chemotherapy regimens [8]. The estimated median overall survival time in advanced MOC (FIGO stages III or IV) is 12–33 months [3,9].

Mucinous ovarian carcinoma patients should have surgery. MOC is characterized by a large, multilocular neoplasm. If the neoplasm ruptures during surgical resection, mucus will penetrate the pelvic and abdominal cavities and adhere to the surfaces of other organs. The slow aspiration of mucus prolongs the operative time. MOC mucus overflow can lead to peritoneal implantation, long-term recurrence and metastasis, and a poor prognosis [10]. Normal saline, urokinase, glucose at various concentrations, low molecular dextran, and other solutions have been used in clinical practice to dissolve the pelvic and abdominal disseminated mucus, albeit the effect is not optimal [4]. Therefore, cyst rupture should be avoided at all costs during the surgical resection of mucinous tumors. It is still a dilemma to clear the mucus during surgery.

The differential diagnosis and pathological classification of mucinous ovarian cancer present further challenges. According to the growth pattern, the World Health Organization divided mucinous ovarian cancer into two categories in 2014, namely, the expansile subtype and the infiltrative subtype [11]. The distinction between the expansile and infiltrative subtypes is clinically important in stage I disease because it may influence indications for staging lymphadenectomy or adjuvant chemotherapy. The following sections of this review will cover the differential diagnosis of primary and secondary MOC.

Most MOCs diagnosed in the early stage usually have a good prognosis postoperatively. Patients with advanced MOCs have a very poor survival which may be attributed to the insensitivity to conventional platinum-based chemotherapy [3,10]. Selecting an efficient chemotherapy regimen is another challenging aspect. Because MOCs and gastrointestinal tumors have comparable pathological and molecular features, retrospective studies have found that MOC patients benefit from empirical gastrointestinal chemotherapy regimens [[12], [13], [14], [15]]. There was only one randomized trial (Gynecologic Oncology Group trial 0241) that compared capecitabine and oxaliplatin (a gastrointestinal chemotherapy regimen) versus carboplatin and paclitaxel for the treatment of MOCs, which has been unfortunately terminated prematurely because of the rarity of MOC [16]. Therefore, the exact cause of the low response rate of MOCs to the conventional platinum-based combination chemotherapy regimen remains unclear. Prospective clinical studies in MOC patients are challenging to conduct. So far, significant efforts have been made to investigate the pathological and molecular features of MOCs to identify new therapeutic strategies. Mucins are a unique pathological feature of mucinous carcinomas, and they are expected to be a potential therapeutic target for advanced MOCs [3,17].

## 3 Mucins and the development of MOC

Mucus substance is a complex mixture of hydrocarbon macromolecules, which is divided into three types: polysaccharides, proteoglycans, and glycoproteins. The mucus secreted by epithelial tissues mostly belongs to glycoprotein. Ovarian mucinous tumors (OMTs) develop from epithelial cells with distinct biological characteristics, such as the presence of a large amount of mucus in the intracellular and/or extracellular microenvironment. Mucus is a glycoprotein mainly consisting of mucins, with acidic mucins (mucin sulfate) dominating MOC.

Mucins are a class of glycoproteins with a large molecular weight, which are characterized by high O-glycosylation and continuous repetitive peptide sequences. They are widely distributed in the human body and have a variety of functions. Mucins are classified into two types based on their forms and functions, namely secreted mucins and membrane-bound mucins. Secreted mucins are gel-forming mucins found on the mucosal or cell surface that consist of MUC2, MUC5AC, MUC5B, MUC6, and MUC19, whereas membrane-bound mucins reside on the surface layer of epithelial cells. Currently, MUC1, MUC3, MUC4, MUC12, MUC13, MUC15, MUC16, MUC17, MUC20, MUC21, and MUC22 are found in the transmembrane form.

During tumorigenesis, mucins are closely linked with tumor cell behaviors and signal transduction [18]. MUC1, the first mucin identified, has been found in tumor cells to exhibit upregulated expression, abnormal glycosylation, and nonpolar distribution. MUC1 regulates tumor cell proliferation, epithelial-mesenchymal transition (EMT), and epigenetics, a vital tumor regulator. The therapeutic and diagnostic potential of MUC1 in tumors has been demonstrated [19]. MUC1 is upregulated in EOC tissues compared with adjacent tissues and positively correlated with tumor staging. Its identification assists in evaluating disease progression in EOC patients [20,21]. The positive expression of MUC1 detected by immunohistochemistry is also found upregulated in MOC tissues [[22], [23], [24]]. MUC16 has been used as an ovarian cancer biomarker for several years. MUC16 overexpression has been associated with tumor progression, metastasis, and a poor prognosis, albeit the exact mechanism is unknown. MUC16 regulates tumor cell proliferation by mediating glucose transporter 1 (GLUT1). MUC16 regulates glucose absorption in EOC cells by controlling GLUT1, increasing glycogen production and the energy available for tumor growth [25]. Mesothelin is a crucial regulator in the multistep process of ovarian cancer peritoneal spread [26], which is stimulated by MUC16 via binding to mesothelial cells, allowing tumor cells to adhere to mesothelial cells [27].

OMTs, unlike serous EOCs, progress from benign to borderline to malignant. Mucin expression intensities and types vary greatly throughout tumor staging and can be used to predict disease progression (Table 1). Most studies found that MUC2 expression increased from benign to borderline to malignant OMTs. MUC2 is believed to promote the development of primary MOCs. It may be a helpful indicator of MOC clinical outcomes [25,[28], [29], [30]]. Simultaneously, the positive expression of MUC5AC differs. MUC5AC was expressed positively in all stages of OMTs and was commonly expressed in MOCs [29,31]. Another mucin that has received increased attention in OMTs is MUC6. The expression level of MUC6 decreased with an increase in tumor grade [30]. Weak expression of MUC6 was closely linked with the development of mucinous tumors [25]. Positive expression of MUC13 in MOCs has also been confirmed [32]. In addition to MUC16, MUC13 may be useful in detecting some subtypes of nonserous ovarian carcinomas and early-stage ovarian carcinomas (stages I and II) [33]. The expression of MUC1 and MUC16 has been less studied in OMTs of different pathological types [23,25,34]. In conclusion, mucins play an important role in the occurrence and progression of tumors. Their positive expression in MOC may be associated with tumor grade, tumor stage, and, ultimately, prognosis. However, all the articles included in this review described retrospective studies that mostly used immunohistochemical staining to compare mucin expression levels. In comparison with the commonly used MUC16, other mucins are rarely utilized to diagnose and detect OMTs in clinical practice. Basic research and prospective studies are required to investigate the link between mucins and the prognosis of MOC. It has come to our attention that MUC2, MUC5AC, MUC6, and MUC13 appear to be more closely associated with the incidence and development of MOC. In early-stage MOCs, MUC2 and MUC13 show promise as potential predictors. They may be utilized to forecast the progression of mucinous carcinoma in the future.

**Table 1:** Expression profile of mucins in ovarian mucinous tumors.

Protein of Interest	Subtype			Detection methods
	Mucinous adenoma	Mucinous borderline tumor	Mucinous adenocarcinoma	
MUC1				
(Hou et al., 2017)	N/R	N/R	25/30 (83.33%)	IHC,ICC,IP
(Wang and El‐Bahrawy, 2015)	2/9 (22.22%)	3/25 (12%)	6/19 (31.6%)	IHC
**MUC2**				
(Wang and El‐Bahrawy, 2015)	0/9 (0%)	10/25 (40%)	8/19 (42.11%)	IHC
(Albarracin et al., 2000)	0/12 (0%)	5/10 (50%)	7/10 (70%)	IHC
(Hirabayashi et al., 2008)	6/29 (20.69%)	16/29 (55.17%)	15/29 (51.72%)	IHC
(Ohya et al., 2021)	67/104 (64.42%)	38/55 (69.09%)	10/18 (55.56%)	IHC
**MUC5AC**				
(Wang and El‐Bahrawy, 2015)	9/9 (100%)	25/25 (100%)	18/19 (94.8%)	IHC
(Albarracin et al., 2000)	12/12 (100%)	10/10 (100%)	10/10 (100%)	IHC
(Vitiazeva et al., 2015)	4/4 (100%)	N/R	4/4 (100%)	IHC
(Hirabayashi et al., 2008)	6/29 (20.69%)	29/29 (100%)	22/26 (84.62%)	IHC
(Ohya et al., 2021)	104/104 (100%)	55/55 (100%)	18/18 (100%)	IHC
**MUC6**				
(Wang and El‐Bahrawy, 2015)	6/9 (66.67%)	4/25 (16%)	5/19 (26.32%)	IHC
(Hirabayashi et al., 2008)	16/29 (55.17%)	6/29 (20.69%)	11/26 (42.30%)	IHC
(Ohya et al., 2021)	83/104 (79.81%)	34/55 (61.82%)	10/18 (55.56%)	IHC
**MUC13**				
(Chauhan et al., 2009)	N/R	N/R	13/13 (100%)	Tissue microarray IHC,RT-PCR
**MUC16**				
(Vitiazeva et al., 2015)	3/4 (75%)	N/R	4/6 (66.67%)	IHC

*Abbreviation:*IHC,Immunohistochemistry; ICC,immunocytochemistry; IP,Immunoprecipitation; RT-PCR,Reverse.

transcription Polymerase Chain Reaction; N/R, Not reported.

## 4 Mucins contribute to the differential diagnosis of primary and secondary MOC

MOC accounted for 10%–15% of EOC cases in early reports [10,35,36]. However, a central pathological review of OMTs revealed that the vast majority of tumors are metastases from other sites. The gastrointestinal tract is the most common metastatic site of MOC, which can also originate from the breast or cervix, whereas the true primary mucinous cancer of the ovary accounts for only 1%–3% [2,3]. Identifying the primary site of MOC is important for evaluating prognosis and selecting appropriate treatment methods.

Currently, the differential diagnosis methods of primary and secondary MOC mainly include the evaluating clinical symptoms, pathological examinations, and immunohistochemical staining. Primary MOC (PMOC) is characterized by large tumor size (>10 cm), a unilateral ovarian lesion, a normal appendix, the absence of capsular or serosal implants, negative gastrointestinal findings, the absence of extracellular mucins [3]. Furthermore, the existence of benign or borderline components indicate the occurrence of PMOC [37]. Metastatic MOC (MMOC) is featured ovarian surface and hilum involvement, infiltrative stromal invasion, extracellular mucin synthesis, and widespread distribution of signet ring cells [38]. Commonly used markers for immunohistochemical staining include CK7, CK20, CDX2, PAX8, and estrogen/progesterone [3,39]. Several new biomarkers for differential diagnosis have been discovered recently, such as SATB2, claudin 18.2, and mucins [3,40,41]. Studies have found that combinations of different biomarkers could improve the diagnosis of PMOC. The combination of CK7 and SATB2 can distinguish lower gastrointestinal tumors from primary OMTs with great accuracy [41]. Claudin 18.2 was highly expressed in both PMOC and MMOC. The common immunophenotypic characteristics of PMOC, upper gastrointestinal tract-derived MMOC, and lower gastrointestinal tract-derived MMOC were claudin 18.2+/PAX8+/SATB2−, claudin 18.2+/PAX8−/SATB2−, and claudin 18.2−/PAX8−/SATB2+, respectively [40]. Mucin distribution and expression patterns can help in the differential diagnosis of PMOC and MMOC. PMOC is often characterized by high levels of intracellular mucin and low levels of extracellular mucin, whereas MMOC is characterized by high levels of extracellular mucin. Extracellular mucin expression may also correlate with a poor prognosis in PMOC [42,43]. A comparison of mucin expression patterns in PMOC and MMOC tissues by immunohistochemistry can be used in differential diagnosis. Chu et al. evaluated MUC1, MUC2, and MUC6 expression levels in 19 MOC patients from different origins, such as the lower gastrointestinal tract, pancreas, and stomach, and suggested that only MUC2 and MUC6 are helpful in the diagnosis of PMOC [44]. Shin et al. investigated MUC2 and MUC5AC expression in colorectal and mucinous ovarian cancers. MUC2 positivity was found in 51% of colorectal cancers and 0% of mucinous ovarian cancers, respectively. MUC5AC, on the other hand, has a frequency of 2.4% and 50% [45]. In ovarian, pancreatic, biliary, esophageal, gastric, and colorectal/appendiceal adenocarcinomas, MUC5AC+/MUC1− is typically detected in PMOC tissues, whereas MUC5AC+/MUC1+, MUC5AC−/MUC1+, and MUC5AC−/MUC1− are detected in pancreatic and esophageal, biliary, and gastric and lower gastrointestinal adenocarcinomas, respectively [25]. Ji et al. used immunohistochemistry to examine the positive expression of MUC5AC in PMOC and MMOC tissues. They found that while MUC5AC can help distinguish colorectal cancer from PMOC, it cannot contribute to the differential diagnosis of PMOC and pancreatic mucinous carcinoma [46]. Mucinous carcinomas of the ovary and colon were examined by Chelariu-Raicu et al. using both standard (PAX8, CK20, CK7, CDX2, SATB2, and estrogen/progesterone) and novel (MUC1 and MUC5AC) biomarkers. They found that MUC1 can be used as a new biomarker to differentiate between primary and metastatic mucinous ovarian cancer. In addition, the tumor growth pattern and the PAX8 immunophenotype might represent potential prognostic biomarkers for PMOC [39].

Taking these together, intracellular mucins are dominant in PMOC. MUC1, MUC2 and MUC5AC are more significant than any other mucins for the differential diagnosis of PMOC and MMOC, particularly in gastrointestinal carcinomas. However some mucins are both positively expressed in PMOC and MMOC [47]. The differential diagnosis of MOC requires a combination of other indicators such as CK7, CK20, CDX2, PAX8, SATB2, and claudin 18.2, which may further reduce the misdiagnosis and missed diagnosis of mucinous ovarian cancer.

## 5 Mucins in the treatment of MOC

Mucins are important in treating MOC because they are associated with chemotherapy resistance in mucinous carcinomas and can be used as potential targets for cancer immunotherapy.

### 5.1 Mucins and chemotherapy-resistant of MOC

Chemotherapy resistance is a common feature of MOC, in which, mucins are involved. A systematic review found that overexpressed mucin glycoproteins such as MUC1, MUC4 and MUC16, are associated with the resistance to apoptosis, chemotherapy and radiotherapy in many epithelium-derived malignancies [19]. Carboxyl-terminal MUC16 has been shown to inhibit TRAIL-induced apoptosis and reduce cisplatin sensitivity [48,49]. Overexpression of secreted mucin results in the resistance of the colorectal cancer cell line HT29 to fluorouracil or methotrexate [50]. Breast cancer cells are more sensitive to Herceptin after silencing of MUC1 [51]. Trastuzumab resistance can be overcome in HER-2-positive gastric tumors by silencing MUC1 or MUC4 [52,53].

The following mechanisms are involved in the role of mucins in inducing chemotherapy resistance.

(1) Mucins are glycoproteins with a large number of clustered O-glycans. MUC2, MUC5AC, MUC5B and MUC6 share similar domain organization and structural features. The folded N- and C-terminal domains facilitate the formation of disulfide-mediated polymers. The scaffolds for adding O-linked glycans are the central, repetitive proline-, threonine-, and serine-rich (PTS) regions. The major gel-forming mucins contain multiple copies of a folded, calcium-binding domain, known as CysD, embedded within their PTS regions. The diverse structural elements of gel-forming mucins contribute to forming protective mucus barriers blocking cell accessibility to therapeutic drugs [[54], [55], [56]].
(2) Cytotoxic therapy is intended to induce apoptosis, and resistance to programmed cell death contributes significantly to chemotherapy resistance. Mucin expression may reduce cancer cell sensitivity to genotoxic drugs by inhibiting the apoptotic effect response to DNA damage or physiological stress [46].
(3) Mucins affect drug metabolism. Tumor cell metabolic regulators help improve pancreatic tumor cells' sensitivity to chemotherapy drugs, such as nucleoside analogs (e.g., gemcitabine and 5-FU), particularly nucleoside transporters. MUC4 and hCNT1 are potential new targets for improving pancreatic tumor response to gemcitabine therapy. Changes in nucleotide metabolism are responsible for the role of MUC4 in inducing the resistance to gemcitabine, and MUC4 negatively regulates hCNT1 via the NF-κB signaling pathway [57].
(4) Mucins are associated with cancer stem cells (CSCs). Recurrence is generally caused by the self-renewal ability of CSCs within the tumor. MUC4 overexpression significantly increases CD133-370-positive CSCs in ovarian cancer [58].
(5) EMT, characterized by the loss of cell polarity, downregulation of epithelial markers such as E-cadherin and cytokeratin-18, and upregulation of mesenchymal markers such as vimentin, N-cadherin and MMP-9, is linked with tumor growth, metastasis, and recurrence. Mucins like MUC1, MUC4, and MUC16 initiate the molecular process of EMT [44].

MUC1 and MUC2 have been served as therapeutic targets for chemotherapy-resistant MOC [59,60]. Mucins may be used in specialized vectors to increase tumor sensitivity to specific chemotherapy. A MUC1/let-7i chimera combines MUC1 aptamer with let-7i miRNA in the ovarian cancer cell line OVCAR-3, reversing the chemotherapy resistance to paclitaxel [61]. Similarly, chemotherapy resistance to paclitaxel is reversed by a MUC1/miR-29b chimera [62]. The breakdown of surface mucus in MOC and its effect on the chemotherapy efficacy has been explored. The viability of the MOC cell line OMC685 is significantly inhibited by the treatment of Endo-N-acetylgalactosaminidase resolvase and paclitaxel compared to those only treated with paclitaxel. The effect of paclitaxel on inhibiting cell proliferation has been greatly improved by disrupting the physical protective barrier on the surface of cancer cells through decomposing mucins. Mucins are important in influencing chemotherapy's therapeutic efficacy on MOC [63]. Collectively, mucins are promising targets for overcoming the chemotherapy resistance of MOC.

### 5.2 Mucins and targeted therapy for MOC

Targeted therapy inhibits the growth and spread of cancer cells by targeting cancer-associated molecules [64]. Targeting biomarkers and biological triggers of MOCs have yielded promising outcomes [65]. Some molecular targets of MOCs have been tested in clinical trials, including HER2, WEE1 tyrosine kinase, CA-125, VEGF, and EGFR [17].

Mucins are promising therapeutic targets for ovarian cancer. Because of the rarity of MOC, prospective clinical studies are challenging to conduct. This section summarizes the outcomes of recent clinical trials that used mucoproteins as targets for the treatment of several kinds of ovarian cancer, including advanced and recurrent mucinous ovarian cancer. Clinical trials of mucin-based targeted therapies are listed in Table 2. Gatipotuzumab, a humanized monoclonal antibody, recognizes the tumor-associated mucin-1 (TA-MUC1) carbohydrate-induced epitope and binds TA-MUC1 selectively with high affinity. Gatipotuzumab was found to be effective in clinical trials for the treatment of advanced ovarian cancer but not for the maintenance treatment of recurrent ovarian cancer [[66], [67], [68]]. CVac is a dendritic cell vaccine that targets the MUC-1 glycoprotein. CVac has shown some efficacy in the treating advanced ovarian cancer in various phase II clinical trials [69,70]. Oregovomab, a murine monoclonal antibody with a high affinity for MUC16, stimulates a cytotoxic immune response in the host against CA125-expressing tumor cells. Oregovomab combined with first-line chemotherapy significantly improves progression-free survival (PFS) and overall survival (OS) in advanced ovarian cancer (OC) patients [[71], [72], [73]]. These findings provided the rationale for the phase III trial FLORA-5 (NCT04498117), which evaluated origomumab with chemotherapy in the neoadjuvant setting and in patients with newly diagnosed advanced OC after optimal cytoreductive surgery [74]. Abagovomab, a monoclonal antibody that mimics MUC16, has been shown to stimulate tumor-specific immune responses in preclinical and phase I/II trials. Nine MOC patients were included in a phase III trial comparing abagovomab with placebo as a maintenance therapy, no improvement in recurrence-free survival (RFS) or OS was found for any patient [75]. Nonetheless, simultaneous targeting of multiple tumor antigens has shown promising results in other solid tumor trials, suggesting that it may be a viable option for future trials targeting tumor antigens. The discovery of antibody-drug conjugates (ADCs) has shown promising results for future clinical development in platinum-resistant recurrent ovarian cancer (PROC). Based on the idea of immune bioconjugation, ADCs are featured by the selectively delivering cytotoxic drugs to cancer cells that positively express various antigens [76]. DMUC5754A and DMUC4064A have humanized anti-MUC16 monoclonal antibodies conjugated to the microtubule-disrupting compound monomethyl auristatin E (MMAE). Both showed an acceptable safety profile and signs of anti-tumor effects in treating platinum-resistant recurrent ovarian cancer [77,78]. REGN 4018, a bispecific T-cell-binding antibody that induces T-cell activation and kills MUC16-expressing tumor cells *in vitro*, has been shown effective in inhibiting the growth of intraperitoneal ovarian tumors. REGN 4018 combined with a PD-1 (anti-programmed cell death 1) antibody and anti-VEGF can be more effective [79,80]. BiTEs-REGN-5668 (MUC16/CD28) study of the combined treatment of REGN4018 or PD-1 monoclonal antibody for recurrent OC has also entered phase II clinical trials (ClinicalTrials.gov Identifier:NCT04590326). Chimeric antigen receptor T (CAR-T) cell technology has shown benefits in some solid tumors as a new type of immunotherapy. Chekmasova et al. used chimeric antigen receptor T-cell (CAR-T) therapy to successfully eradicate mouse OC targeting the MUC16 antigen [81]. CAR-T cells effectively prolong the survival time in mice with OVCAR-3 tumors, suggesting the extent of therapeutic efficacy. Dual-target CAR-T cells are found to be 2–4 times more successful than single CAR-T cells in prolonging the survival [82].

**Table 2:** Mucins and targeted therapy for ovarian cancer in clinical trials.

Authors	Year of Publication	Therapeutic agents (molecular target)	Type of Study	Types of cancers eligible	Total Patient	Pathological types	Key findings
(Fiedler et al., 2016)	2016	Gatipotuzumab （MUC1）	Phase I	Treatment of advanced ovarian carcinomas	20	N/R	The ORR was 40%. 5% patients achieve CR and 35% SD.
(Ledermann et al., 2022)	2022	Gatipotuzumab (MUC1)	Phase II	Maintenance therapy of patients with recurrent epithelial primary ovarian, fallopian tube, or primary peritoneal cancer	216	N/R	No improvement in PFS or OS observed.
(Gray et al., 2014)	2014	Cvac （MUC1）	Phase II	Treatment in stage III or IV epithelial ovarian cancer who obtained a complete response to standard first (CR1) or second-line chemotherapy (CR2)	63	N/R	Improvement in PFS in 20 patients in CR2.
(Mitchell et al., 2014)	2014	Cvac （MUC1）	Phase II	Treatment in patients with epithelial ovarian, fallopian tube, or primary peritoneal cancer	28	Serous - 22 Endometrioid - 1 Endometrioid and clear cell - 1 Other - 2	Four patients showed CA125 response or stabilization (2 patients with major responses, 1 minor response, 1 stabilization).
(Berek et al., 2004)	2004	Oregovomab （MUC16）	Phase III	Maintenance therapy of patients with stage III or IV ovarian cancer	145	Serous - 89 Endometrioid - 14 Other - 42	Prolonged time to relapse 13.2 months for the oregovomab group in the successful front-line therapy population.
(Braly et al., 2009)	2009	Oregovomab （MUC16）	Phase II	Treatment of advanced ovarian cancer	40	Serous - 31 Mucinous - 2 Other - 7	33 patients achieved CR to surgery-carboplatin-paclitaxel-oregovomab.
(Brewer et al., 2020)	2020	Oregovomab （MUC16）	Phase II	Front-line chemo-immunotherapy with carboplatin-paclitaxel using oregovomab indirect immunization in advanced ovarian cancer	97	Mucinous - 2 Serous - 86 Endometrioid - 6 Clear cell - 2 Other - 1	Significant improvement in PFS and OS. Prolonged PFS 29.6 months for the oregovomab group. OS has not yet been reached.
(Sabbatini et al., 2013)	2013	Abagovomab （MUC16）	Phase III	Maintenance therapy of patients with stage III-IV epithelial ovarian, fallopian tube, or primary peritoneal cancer	888	Serous - 726 Endometrioid - 57 Mucinous - 9 Other - 91	No improvement in RFS or OS was observed.
(Liu et al., 2016)	2016	DMUC5754A (ADC-MUC16)	Phase I	Treatment in patients with platinum-resistant recurrent ovarian cancer	66	N/R	Two patients had unconfirmed PR. Six patients had SD lasting >6 months.
(Liu et al., 2021)	2021	DMUC4064A (ADC-MUC16)	Phase I	Treatment in patients with platinum-resistant recurrent ovarian cancer	65	N/R	The clinical benefit rate was 42%. 27 patients had CR, or PR or SD lasting ≥6 months.

*Abbreviation:*Gatipotuzumab,a humanized monoclonal antibody recognizing MUC1; Cvac,a dendritic cell vaccination targeting MUC-1; Oregovomab,a murine monoclonal antibody recognizing MUC16; Abagovomab,a monoclonal antibody recognizing MUC16; DMUC5754A/DMUC4064A,a humanized anti-MUC16 monoclonal antibody conjugated to MMAE; ORR, objective response rate; PFS,progression-free survival; RFS, relapse free survival; OS, overall survival; CR, complete response; CR1, complete response to first chemotherapy; CR2, complete response to second-line chemotherapy; PR, partial response; SD, stable disease; N/R, not reported.

Although mucins' therapeutic efficacy and safety profile in OC patients has been extensively validated in many clinical trials, their clinical use of MOC is limited. Analyses of the results of trials of pan-ovarian cancer treatment in reviews may help doctors make informed decisions about off-label treatment for mucinous ovarian cancer. Studies regarding uncommon tumors are likely to require a multinational effort and may be challenging to carry out. Moreover, mucins appear effective in the targeted therapy but ineffective in the maintenance therapy on OC patients. Simultaneous targeting of multiple tumor antigens has shown promising results in MOC, which may be a reasonable avenue for future trials.

## 6 Conclusions

Mucins typically expressed in MOC have important implications in the development, differential diagnosis, chemotherapy resistance, and immunotherapy of MOC. MUC2, MUC5AC, MUC6, and MUC13 appear to be more closely linked to the occurrence and development of MOC. High-level intracellular mucins characterize primary MOC. MUC1, MUC2 and MUC5AC are dominant in MOC and can be used in the differential diagnosis of PMOC and MMOC, particularly in gastrointestinal carcinomas. The correlation between mucins and chemotherapy resistance in MOC was also analyzed. MUC1 and MUC16 are potential molecular targets for OC treatment. Due to the low prevalence of MOC, it is challenging to perform extensive research and promote clinical practice. The most recent research on mucins and targeted therapy focuses on ovarian carcinomas other than MOC. This review highlights the role of mucins in MOC. Future research is needed to examine the potential of mucin inhibitors in treating MOC, and targeting several tumor antigens simultaneously would be a good idea.

## Author contribution statement

All authors listed have significantly contributed to the development and the writing of this article.

## Data availability statement

Data included in article/supp. material/referenced in article.

## Declaration of competing interest

The authors declare that they have no known competing financial interests or personal relationships that could have appeared to influence the work reported in this paper.
